# Integrating Signaling Pathways with Transcription Factor Networks—On the Trail of Sisyphus?

**DOI:** 10.3390/biom14081015

**Published:** 2024-08-16

**Authors:** Kostas A. Papavassiliou, Athanasios G. Papavassiliou

**Affiliations:** 1 First Department of Respiratory Medicine, “Sotiria” Hospital, Medical School, National and Kapodistrian University of Athens, 11527 Athens, Greece; konpapav@med.uoa.gr; 2 Department of Biological Chemistry, Medical School, National and Kapodistrian University of Athens, 11527 Athens, Greece

In the context of health and disease research, cells use signaling pathways that transduce stimuli from the extracellular environment to modulate intracellular gene expression via the activity of transcription factors and cofactors (coactivators and/or corepressors). Upstream signaling can either potentiate or inhibit transcription factors, primarily through post-translational modifications (mainly phosphorylation), changes in localization (e.g., the masking of nuclear localization signal(s) and cytoplasmic retention by binding to an anchoring protein), alterations in their transcription/translation, or by modifying their ability to access their target genes in an epigenetic manner [1]. A few examples of signaling cascades leading to the upregulation or downregulation of transcription factors are Janus kinase (JAK) signaling, which activates members of the signal transducer and activator of transcription (STAT) family of transcription factors [2]; transforming growth factor beta (TGF-β) signaling, which leads to the activation of the suppressor of mother against decapentaplegic (SMAD) family of transcription factors [3]; Hippo signaling, which targets the TEA domain-containing (TEAD) family of transcription factors [4]; and Wingless-related integration site (WNT) signaling, which induces the activation of β-catenin [5].

Under normal conditions, this intricate molecular process that begins from the cell membrane, continues with intracellular signaling pathways, and ends with transcription factor-dependent gene regulation, allowing cells to acquire and maintain their identity, as well as respond to their dynamic extracellular environment. In the context of disease, abnormal signaling and transcription factor function result in aberrant gene regulation and pathological cell phenotypes. Undoubtedly, signaling pathways and transcription factors are interconnected and should be studied in concert to better understand cellular physiology and pathophysiology. Yet, deciphering the interplay between cell signaling and transcription-factor-mediated gene control still remains a challenge because it is affected by multiple factors and is context-dependent.

In order to draw a full picture of the complex crosstalk between signaling pathways and transcription factors, several recent studies have used comprehensive approaches that integrate multi-omics data, including genomics, transcriptomics, epigenomics, proteomics, phosphoproteomics, and metabolomics. These systematic multi-omics studies have brought new insights into the interaction between signaling pathways and transcription factor networks that advance the current knowledge on the gene regulatory landscape. Importantly, multi-omics data reveal biologically and clinically relevant mechanisms, therapeutic targets, and biomarkers that can be leveraged in the clinic for patient stratification and treatment. For example, Mun et al. recently conducted a study whereby proteomics and post-translational modification data were integrated with transcriptional profiling, uncovering the molecular heterogeneity of early-onset gastric cancer [6]. In another study, Wang et al. reported a multi-omics method based on interactions among proteomics, phosphoproteomics, and transcriptomics that identified numerous oncogenic drivers, including kinases and transcription factors, in glioma [7]. In the research field of early human development, Alanis-Lobato et al. provided a multi-omics approach to predict gene regulatory networks in early human embryos [8]. In the context of human physiology, Yan et al. generated multi-omics data related to transcriptional regulation in response to high-altitude hypoxia [9]. Additionally, a study by Li et al. developed a multi-omics method to unravel the gene expression networks associated with the transcription factor activator protein 1 (AP-1) [10]. In a recent study by He et al., an integrative analysis of multi-omics data unveiled a drug-resistance mechanism involving the transcription factor Rb in hepatocellular carcinoma [11]. Similarly, using muti-omics profiling, Lu et al. discovered key signaling pathways that are modulated by the transcription factor STAT3 in ovarian cancer [12]. These studies, along with several others, underline the important knowledge that can be uncovered by integrating data from multiple molecular layers.

For a dynamic relationship between signaling cascades and transcription factor networks to develop in a biological system, we must be aware of all aspects of -omics since genomics, transcriptomics, epigenomics, and proteomics are deeply interconnected. With the aid of artificial intelligence (AI), comprehensive multi-omics data analyses will revolutionize our understanding of cell signaling and gene regulation in both healthy and diseased states.
